# Folding–unfolding asymmetry and a *RetroFold* computational algorithm

**DOI:** 10.1098/rsos.221594

**Published:** 2023-05-03

**Authors:** Sergey Shityakov, Ekaterina V. Skorb, Michael Nosonovsky

**Affiliations:** ^1^ Infochemistry Scientific Center (ISC), ITMO University, 9 Lomonosova Street, St. Petersburg 191002, Russia; ^2^ College of Engineering and Applied Science, University of Wisconsin-Milwaukee, Milwaukee, WI 53211, USA

**Keywords:** Trp-cage, folding, unfolding, *RetroFold* algorithm, molecular dynamics

## Abstract

We treat protein folding as molecular self-assembly, while unfolding is viewed as disassembly. Fracture is typically a much faster process than self-assembly. Self-assembly is often an exponentially decaying process, since energy relaxes due to dissipation, while fracture is a constant-rate process as the driving force is opposed by damping. Protein folding takes two orders of magnitude longer than unfolding. We suggest a mathematical transformation of variables, which makes it possible to view self-assembly as time-reversed disassembly, thus folding can be studied as reversed unfolding. We investigate the molecular dynamics modelling of folding and unfolding of the short Trp-cage protein. Folding time constitutes about 800 ns, while unfolding (denaturation) takes only about 5.0 ns and, therefore, fewer computational resources are needed for its simulation. This *RetroFold* approach can be used for the design of a novel computation algorithm, which, while approximate, is less time-consuming than traditional folding algorithms.

## 1. Introduction

**Figure 1.**
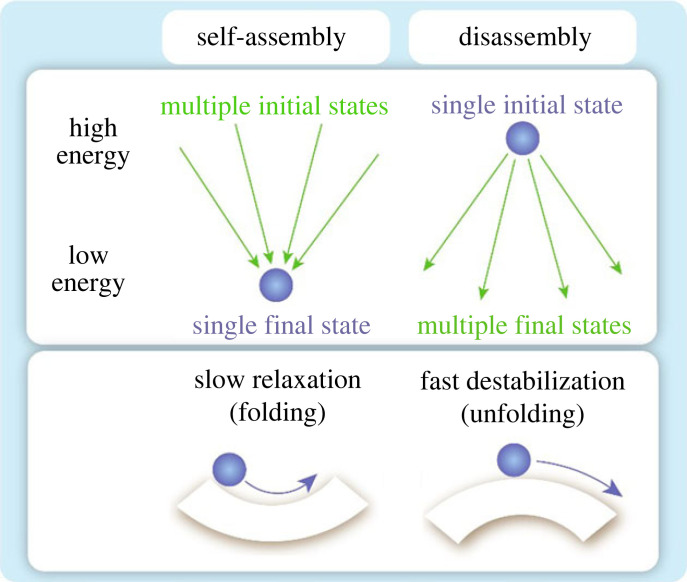
Schematic showing self-assembly (from multiple initial states to a single final state) and disassembly (from a single initial state to multiple final states), and corresponding energy landscapes (slow relaxation or decaying oscillations leading to the self-assembled configuration and fast destabilization during disassembly).

Molecular self-assembly including protein folding is a highly non-equilibrium dynamic process, which is still not well understood and requires a lot of computational resources for its simulation. During self-assembly, the physical system passes through a complex trajectory in the phase space from an initially disordered high-energy macrostate (with many microstates Ω and thus with high entropy S=klnΩ) to a final ordered low-energy macrostate (with a single microstate and low entropy). The process involves dissipation, which leads to relaxation and wandering through intermediate metastable states (figure 1).

As opposed to that, during spontaneous disassembly or disintegration, the system passes from an initially ordered high-energy and low-entropy macrostate to a final disordered low-energy and high-entropy state. In both cases, the energy is minimized, while the entropy decreases during self-assembly and increases during disassembly. In accordance with the Gibbs formula, Δ*G* = Δ*H*−*T*Δ*S*, the enthalpy term Δ*H* prevails over the entropic term, *T*Δ*S*, for low temperatures. Consequently, self-assembly usually cannot be presented as a time-reversed process of disassembly since there is no symmetry between these two processes. The entropy and enthalpy contributions to the coil–globule phase transition of proteins are reviewed in [1].

Self-assembly is often an exponentially decaying process, since energy relaxes due to dissipation, while the system is drifting slowly through metastable states before reaching the most stable configuration. On the other hand, during fracture, driving forces are often equilibrated by damping leading to a constant-rate process. Therefore, to relate the energy release during fracture with the exponentially decaying energy release during self-assembly, different temporal scales should be considered.

Protein folding typically takes dozens of nanoseconds for initial folding by hydrophobic forces and then on the order of microseconds for reaching the stable configuration through relaxation and forming stable intramolecular bonds between adjacent amino acids (AAs). The unfolding usually takes several orders of magnitude less time, on the order of dozens of nanoseconds.

Unfolding and denaturation of proteins have been studied for more than 60 years [2–9], and various mechanisms, such as cold denaturation [5] and pressure-induced denaturation [6], have been investigated. The entropic nature of folding has been investigated as well; thus, Kellermayer *et al*. [7] showed that the force required to stretch a single molecule behaves as a highly nonlinear entropic spring. Force hysteresis arises from a difference between the folding and unfolding kinetics. Intermediate states between the folded and the completely unfolded conformation have been found [8].

During folding, most protein molecules pass from a one-dimensional linear primary structure to a unique three-dimensional native ternary state. Various computational methods can be applied to model folding including the molecular dynamics (MD) and Monte Carlo (MC) simulation, as well as machine-learning (ML) methods [10–12]. The MD allows visualization of the transitional states (TS) structures dynamically, but it is very slow and time-consuming for a large number of rotational degrees of freedom. The MC (e.g. *RoseTTAFold*) simulation is faster than the MD; however, a crystallographically obtained reference structure is needed for this method. New ML methods, such as the popular *AlphaFold* [10], are relatively fast and precise; however, they are not applicable to TS analysis.

Since modelling protein folding remains a challenging computational task consuming significant resources, various heuristic approaches to folding modelling have been proposed, including scaling and topological methods [13–18]. In the present paper, we suggest a mathematical transformation that presents the assembly–disassembly process as quasi-reversible. We will apply it to the folding–unfolding transition to suggest a simplified method of folding modelling as a time-reversed unfolding.

## 2. A mathematical log transition for self-assembly/disassembly asymmetry

In this section, we consider a model one-dimensional system consisting of a particle with the mass *m* travelling in a potential field with dissipation. This is a simplified linearized model of self-assembly and disassembly. For the potential field with a global minimum, the particle oscillates with exponentially decaying amplitude and reaches the equilibrium point slowly. This is similar to the self-assembly of a complex system reaching the unique ordered self-assembled state. This is also similar to the folding of a protein molecule reaching its native (globular) state. On the other hand, for the potential field with a global maximum, the particle's motion is unstable, and it reaches the disordered state within a short time. This is similar to the disassembly of a complex system or to the unfolding of a protein molecule. Another possible analogy is the mixing and separation of two types of elements or substances.

Let us consider the motion of a particle in a potential field with dissipation with the Lagrange function, $L(x,\dot{x})$, energy, $E(x,\dot{x})$ and dissipative function, $Q(\dot{x})$, given by [19]

2.1 $$L(x,\dot{x})=\frac{m}{2}{\dot{x}}^{2}-\frac{k}{2}x^{2},\;E(x,\dot{x})=\frac{m}{2}{\dot{x}}^{2}+\frac{k}{2}x^{2},\;Q(\dot{x})=\frac{\beta }{2}{\dot{x}}^{2},$$

where *m*, *k* and *β* are the mass, elastic spring constant and viscous dissipation, respectively. The quadratic potential energy is the simplest energy profile with its minimum corresponding to the state of equilibrium. The equation of motion is supplied by the Lagrange equation

2.2 $$\frac{d}{dt}\frac{\partial L}{\partial \dot{x}}-\frac{\partial L}{\partial x}=-\frac{\partial Q}{\partial \dot{x}}\;\mathrm{or}\;m\ddot{x}+\beta \dot{x}+kx=0.$$

The solution is obtained by the standard substitution of $X=e^{zt}$ yielding the roots of the characteristic equation

2.3 $$z=-\frac{\beta }{2m}\pm \sqrt{{\left(\frac{\beta }{2m}\right)}^{2}-\frac{k}{m}}=\left(-\zeta \pm \sqrt{\zeta^{2}-1}\right)\omega_{0},$$

where $\omega_{0}=\sqrt{k/m}\;$ is the natural frequency, $\zeta =(\beta /\sqrt{km})<1$ is the damping ratio and $\zeta \omega_{0}$ is the damped frequency.

For k>0, the solution is decaying. The underdamped solution (ζ<1) corresponds to complex conjugate roots $z=\left(-\zeta \pm i\sqrt{1-\zeta^{2}}\right)\omega_{0}$ and damped oscillations

2.4 $$x(t)=x_{0}e^{-\zeta \omega_{0}t}\cos\left(\sqrt{1-\zeta^{2}}\omega_{0}t-\phi \right),$$

where ϕ is the phase angle. The overdamped (relaxation without oscillations) solution (ζ>1) corresponds to two negative real roots and it is given by

2.5 $$x(t)=\;x_{0}\left(ce^{\sqrt{\zeta^{2}-1}\omega_{0}t}+e^{-\sqrt{\zeta^{2}-1}\omega_{0}t}\right)e^{-\zeta \omega_{0}t}.$$

In both cases, the total energy is decaying exponentially by

2.6 $$E(t)=E_{0}+(E_{1}-E_{0})e^{-\zeta \omega_{0}t}.$$

For the unstable equilibrium, we will consider the potential field $E(x,\dot{x})=(m/2){\dot{x}}^{2}-(K/2)|x|$. Unlike the quadratic term in equation (2.1), the |x| term corresponds to the non-equilibrium down-the-hill kinetics, which is inherent to protein folding and unfolding [20]. The driving force is asymptotically equilibrated by friction, $K=\beta \dot{x}$, so the energy is changing linearly with time as

2.7 $$E(t)=\frac{m}{2}{\left(\frac{K}{\beta }\right)}^{2}-\frac{K^{2}}{2\beta }t.$$

Note that the stable and unstable solutions cannot be converted to each other by just reversing time due to different time scales of exponential decay and linear growth. To overcome this, one can introduce the coordinate transformation ϑ=T(t) so that

2.8 $$t=\Delta te^{-\zeta \omega_{0}\Delta t\vartheta },$$

where Δt is the total integration time. Equations (2.7) and (2.8) combined yield the equation

2.9 $$E(\Delta t\vartheta )=\frac{m}{2}{\left(\frac{K}{\beta }\right)}^{2}-\frac{K^{2}\Delta t}{2\beta }e^{-\zeta \omega_{0}\Delta t\vartheta },$$

which is structurally similar to equation (2.5).

Thus, the coordinate transformation $\left(x,t)\to (x,\Delta te^{-\zeta \omega_{0}\Delta t\vartheta }\right)$ essentially reduces the self-assembly to the time reversal of disassembly; however, it misses certain details, such as the oscillatory behaviour of the underdamped solution.

A computational method can be suggested when the final self-assembled state of the system (for example, the native structure of a folded protein) is known; however, information about the stages and trajectory of the self-assembly process is needed. In that case, the disassembly can be simulated (for example, unfolding of a protein) and the above-described procedure applied with time reversal. Such an algorithm will likely miss some details, such as oscillating between the metastable states; however, it still can provide a general grasp of the self-assembly trajectory at a computational cost much lower than the direct simulation of self-assembly. We call this the *RetroFold* method as an analogy with the retrosynthesis in organic chemistry, which is considered the reversed synthesis.

## 3. Case study: molecular modelling of the Trp-cage protein

**Figure 2.**
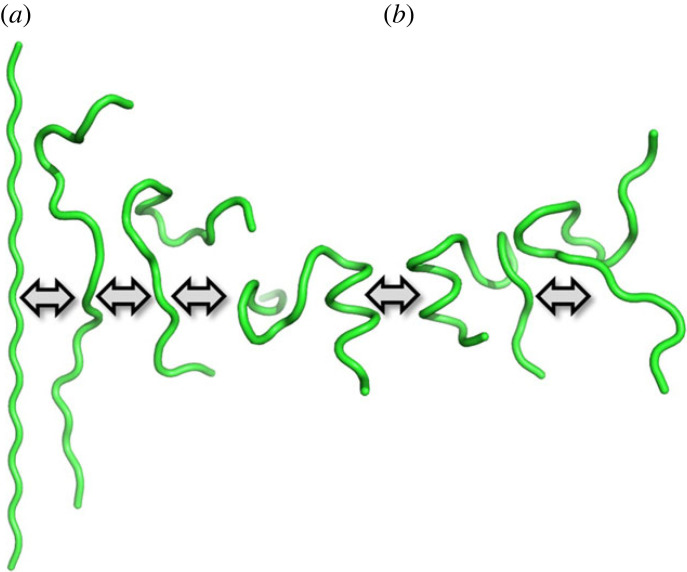
Schematic representation of (*a*) folding and (*b*) unfolding mechanisms for Trp-cage molecule depicted as a tube cartoon [13]. The cartoon models were generated using the PyMOL molecular graphics system (Schrödinger, Inc.).

Trp-cage is the smallest known protein, containing only 20 AA residues, and sometimes it is considered a polypeptide. The name Trp-cage is given due to the central role of the Trp burial in the hydrophobic core and the cage-like shape of the globular structure. The AA sequence of the Trp-cage is Asn-Leu-Tyr-Ile-Gln-Trp-Leu-Lys-Asp-Gly-Gly-Pro-Ser-Ser-Gly-Arg-Pro-Pro-Pro-Ser (NLYIQWLKDGGPSSGRPPPS). Since Trp-cage shares several features with larger globular proteins, it often serves as a model system for various computational studies, including MD simulations analysing protein folding (figure 2). Some observations suggest a two-step folding, while others indicate the presence of intermediate states [13,21,22]. The MD folding simulations demonstrated thermodynamic and kinetic features similar to globular proteins [23].

A two-step mechanism with an intermediate metastable state was found by Zhou [24]. According to this study, two partial hydrophobic cores are separated by an essential salt bridge between residues Asp-9 and Arg-16 near the centre of the molecule [25]. The original value of the melting point was reported as *T_m_* = 42°C; however, mutations in the helical portion of the protein (the replacement of Leu, Ile, Lys or Ser residues by Ala) result in the increase of the melting point to *T_m_* = 64°C from [26]. According to Barua *et al*. [26], the Y3/P19 staple interaction defines the 18-residue folding motif. Along with the Trp burial, this motif is essential for the core formation, unlike the specific Pro/Trp interactions. Other stabilizing features that have been identified include a solvent-exposed Arg/Asp salt bridge (0.81–1.43 kcal mol^−1^) and a buried H-bonded Ser side chain (≈2.39 kcal mol^−1^) [26].

We conducted the MD simulation of both folding and unfolding using the Trp-cage AA sequence with an extended initial conformation built by the LEaP module of AMBER [13,27]. The linear conformation of this protein was designed using Avogadro software [28]. The three-dimensional molecular structure (PDB ID: 1L2Y) of Trip-cage was determined by the nuclear magnetic resonance (NMR) method in the solution as a set (*n* = 38) of stable conformations obtained from the RCSB Protein Data Bank.

The MD simulations included the following phases: minimization (500 cycles), heating (50 ps), and equilibration (production) at 325 K (800 ns) for folding and at 473 K (5 ns) for unfolding according to the standard protocols published elsewhere [13,29,30].

Our MD simulations were fully unrestrained and carried out in the canonical ensemble using the SANDER module available for Linux/Unix. The Berendsen thermostat was implemented for temperature control and the SHAKE algorithm to constrain the length of covalent bonds, including the hydrogen atoms [31]. The ff99 force field was used as it was previously employed for similar modelling [32]. Solvation effects were incorporated using the Generalized Born model, as implemented in AMBER [33]. The Rosetta crystallographic refinement protocol was implemented to assess the conformational stability of the folded and unfolded protein conformations obtained from the previous MD simulations [13,34–37].

**Figure 3.**
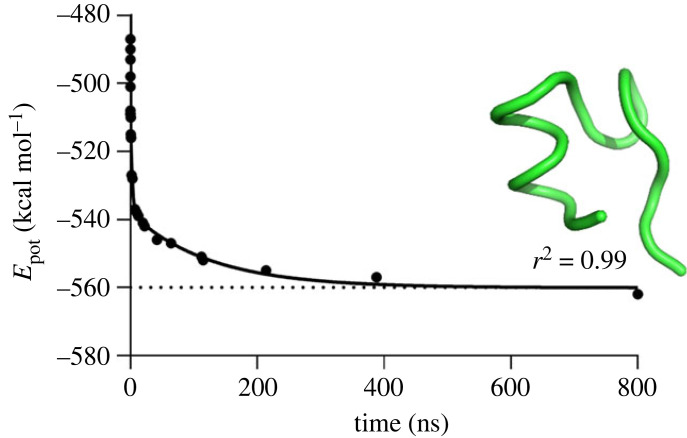
MD simulation of the energy profile for Trp-cage molecule folding showing the potential energy versus simulation time. The 800 ns conformation of Trp-cage is depicted as a tube cartoon model and coloured in green.

The folding process involved two phases: the heating stage, which lasted less than 50 ps, and the equilibration stage, which lasted for 800 ns (figure 3). During the heating phase, the molecule underwent significant structural changes due to the heating disturbances. The root-mean-square deviation (RMSD) of the molecule from its exact final reference native shape (from experimental data obtained by the structural analysis) also undergoes significant fluctuations during folding. During the heating stage, the value of the RMSD was between 16 Å and about 7 Å, and it further decreased during the equilibration. The folding is initiated at the very compliant and flexible C-terminus of the molecule; consequently, the complete folding requires a much longer time of about 4 µs, considering that the RMSD value of 0.56 Å (close to the ideal 0.5 Å) was found with the *AlphaFold2* algorithm [38].

The potential energy of the molecule, *E*_pot_, was used as a measure of folding. The potential energy decreased from *E*_pot_ = −487 kcal mol^−1^ at the beginning of the equilibration stage (*t* = 50 ps) to *E*_pot_ = −562 kcal mol^−1^ at the end of the equilibration stage (*t* = 800 ns), and the decrease is fitted well by an exponential approximation. Note that the rates of folding and unfolding are sensitive to the temperature; however, both processes are qualitatively different, as folding is dominated by dissipative forces and exponential decay, while unfolding is dominated by non-exponential driving forces.

**Figure 4.**
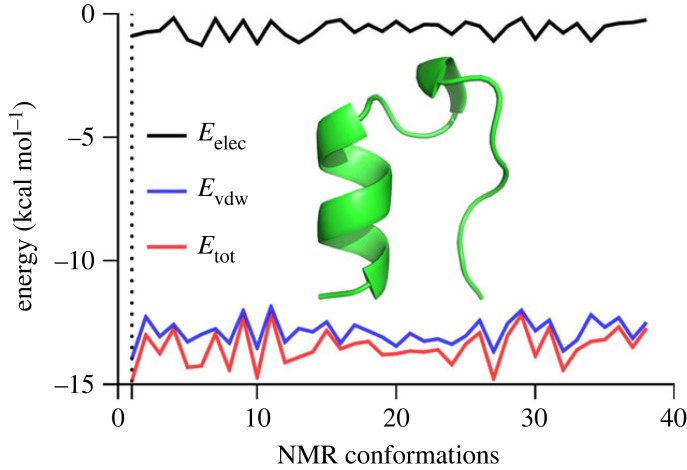
Stability analysis of the 38 NMR conformations of the Trp-cage protein. The most stable first conformation based on the energy of the electrostatic and Van der Waals interactions and the total energy was selected. The reference structure as the first conformation is shown as a cartoon model and coloured in green [13]. The minimal-energy NMR conformation of Trp-cage is depicted as a cartoon model and coloured in green.

The unfolding was modelled with a similar MD simulation protocol starting from the native globular configuration at the temperature of *T* = 498 K. The high temperature was used according to the standard protocol [39]. The most stable conformation (*E*_tot_ = −14.84 kcal mol^−1^) out of 38 different conformations obtained by NMR was used as a starting point for unfolding (figure 4).

**Figure 5.**
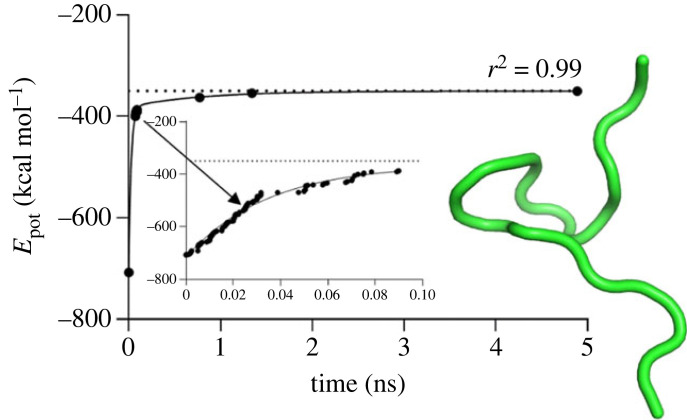
MD simulation of the energy profile for the Trp-cage molecule unfolding showing the potential energy versus simulation time. The 5 ns conformation of Trp-cage is depicted as a tube cartoon model and coloured in green. The energy threshold (−350 kcal mol^−1^) is depicted as dotted lines.

The potential energy showed an almost linear increase at the initial stage from *E*_pot_ = −707.6 kcal mol^−1^ at *t* = 0 ps, up to *E*_pot_ = −478.4 kcal mol^−1^ at *t* = 31.8 ps. Following that, the increase was almost exponential approaching *E*_pot_ = −350 kcal mol^−1^ at about *t* = 1.5 ns, with the unfolding completed before *t* = 5.0 ns (figure 5).

It is observed from this data that folding and unfolding are very asymmetric processes, however the procedure suggested above has been applied. First, the rate of unfolding at the almost linear stage was calculated as 16.25 kcal mol^−1^ ps^−1^. The potential energy was normalized using the initial and the final values of *E*_pot_ = −707.6 kcal mol^−1^ and *E*_pot_ = −350 kcal mol^−1^, respectively, while the time was normalized by dividing by the total simulation time of Δ*t* = 5.0 4 ns, so that the point *E*_pot_ = −478.4 kcal mol^−1^ at *t* = 31.8 ps (or at *τ* = 0.0318/5.0 = 0.0065) corresponded to *ε* = (−478.4−(−707.6))/(−350−(−707.6)) = 0.6418, and the non-dimensional rate of $\zeta \omega_{0}\Delta t=(0.6418/0.0065)=98.74$ was obtained.

Following that, the time variable change or $\vartheta =\ln\tau /(-\zeta \omega_{0}\Delta t)$ was applied, so that the initial unfolding time *t* = 0 (*τ* = 0) corresponded to ϑ→∞ (and the globular state), *t* = 31.8 ps (*τ* = 0.0065) corresponded to ϑ=0.051 (the end of the approximately constant-rate unfolding region) and *t* = 5.0 ns (*τ* = 1) corresponded to ϑ=0 (unfolded state).

At the next stage, the reverse time transition was applied, t=ϑΔt, so that non-exponential folding lasted between t=0 and t=25.7 ns, and the exponential folding was approximated by

3.1 $$E_{\mathrm{pot}}=E_{\min}+(E_{\max}-E_{\min})e^{-\vartheta }=-562\;\left(\frac{\mathrm{kCal}}{\mathrm{mol}}\right)+75\left(\frac{\mathrm{kCal}}{\mathrm{mol}}\right)e^{-t/\Delta t}.$$

**Figure 6.**
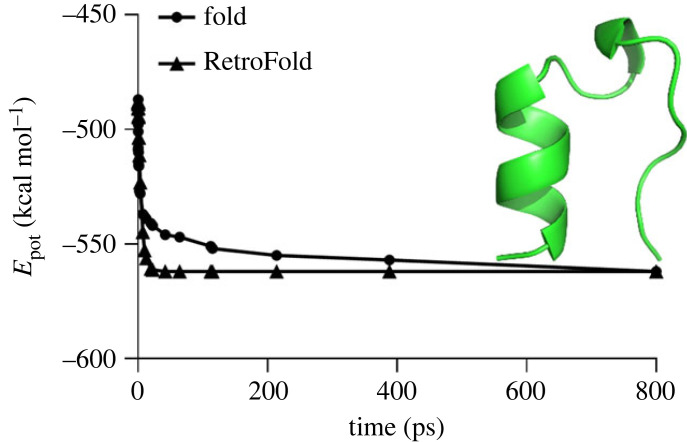
Comparison of the MD simulation of the energy profile for the Trp-cage folding and the simulated data obtained by the *RetroFold* method. The minimal-energy NMR conformation of Trp-cage is depicted as a cartoon model and coloured in green.

When comparing data obtained from the approximate equation (3.1) with the folding simulation data, one finds the maximum error of 4.1% at *t* = 524 ps, while for a time greater than 1 ns, the error is less than 3.5% (figure 6). Thus, one can conclude that the approximate method provides a reasonable approximation to determine the protein structure resembling the NMR model (figure 6).

**Figure 7.**
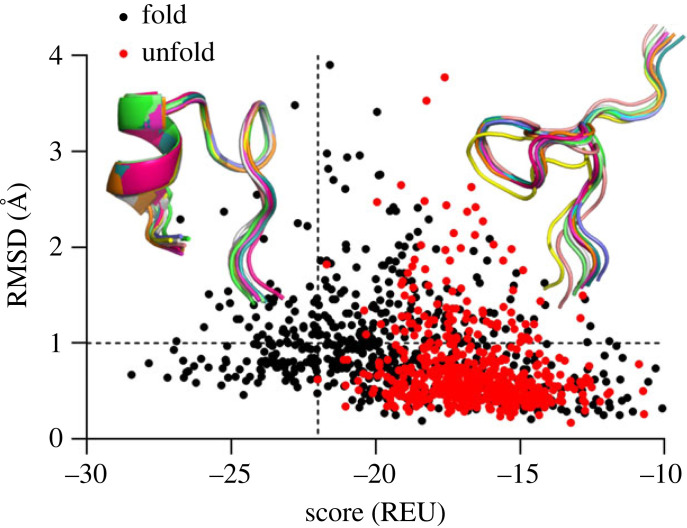
MC analysis of folded and unfolded Trp-cage conformations. The RMSD (1.0 Å) and energy (approx. 22.0 REU) thresholds are depicted as dashed lines. The folded and unfolded reference conformations obtained from MD simulations are depicted as cartoon models and coloured in green.

Finally, the MC analysis was performed using folded and unfolded conformations of Trp-cage mini-protein to assess energy levels for these conformations. It is clear from figure 7 that the folded model of Trp-cage occupies significantly lower energy levels in a range from −28 to −22 REU than its unfolded form (−22 to −10 REU), confirming our previous results. Additionally, the refinement protocol calculated minimal-energy score values of −28.45 REU and −22.0 REU for folded and unfolded states, respectively. All the minimal-energy conformations mainly occurred below the RMSD threshold of 1.0 Å verified by the three-dimensional protein alignment (figure 7), which was previously determined to be lower (0.5 Å) for the NMR models of Trp-cage [13].

## 4. Discussion

The results show a significant asymmetry between folding and unfolding, which can be partially compensated by the transformation T(t) suggested by equation (2.8). The folding–unfolding asymmetry can be related to the inherent non-ergodicity of the folding process. Non-ergodic systems evolve with time, which affects their ability to attain microstates with equal probability, while ergodic systems have no memory of their previous history and attain all available microstates. Ergodicity of a dynamical system implies the equivalence of the phase space averages and time averages. Many biological systems are non-ergodic due to molecular crowding [40], anomalous diffusion, fractal behaviour [14] and many other effects of complex environments typical for biological systems. Non-ergodic behaviour is compensated by considering the mathematical Lamperti transformation [41], which is to some extent similar to the transformation T(t) suggested in the present work, or by introducing the ergodicity defect measure.

Understanding of protein folding involves several difficult problems. While the AA sequence of a protein usually determines its folded (native) structure (the so-called ‘Anfinsen's dogma’ [42]), it is not clear how protein achieves this native structure. The fast kinetics of folding, despite a huge number of possible microstates through which the molecule can pass on its way to the unique native state, is sometimes called the ‘Levinthal paradox’ [43]. The usual explanation of the paradox is that folding is a hierarchical process with many intermediate states between coil and globular proteins, such as secondary and super-secondary structures and domains. These are known as ‘folding intermediates’ [44]. Unfolding does not imply a unique final state, so it takes a much shorter time and also does not necessarily pass through the intermediate state.

While the analysis of the intermediates, such as secondary and super-secondary structures, during unfolding would require the study of larger molecules than the Trp-cage used in our case study, the suggested *RetroFold* approach still provided values of energy within 4.1% implying similar structural configurations at the corresponding stages of the folding and unfolding processes.

## 5. Conclusion

A novel computational method was suggested to simulate self-assembly including protein folding. The method is based on using the disassembly or unfolding data, reversing the time variable, and applying a mathematical log transition, which relates the disassembly (often a constant-rate process) with the self-assembly (often an exponentially decaying process). There is no exact symmetry between the two processes; consequently, the similarity is only approximate. The method was tested for the MD simulations of folding and unfolding of the short Trp-cage protein, and showed agreement within 4.1% or less for the molecular energy profile. Unfolding is more than two orders of magnitude faster than folding (namely, 800 ns for Trp-cage folding versus 5.0 ns for its unfolding) and, consequently, it required two orders of magnitude fewer computer resources to model.

The suggested algorithm will likely miss some details of the folding process, such as oscillating between the metastable states; however, it still can provide a general grasp of the self-assembly trajectory at a computational cost much lower than the direct simulation of self-assembly.

## Data Availability

The dataset is available at Shityakov S, Skorb E, Nosonovsky M (2022), Folding-unfolding asymmetry and a RetroFold computational algorithm, Dryad, Dataset, https://doi.org/10.5061/dryad.2rbnzs7s9 [45].
